# Shotgun metagenomics reveals distinct functional diversity and metabolic capabilities between 12 000-year-old permafrost and active layers on Muot da Barba Peider (Swiss Alps)

**DOI:** 10.1099/mgen.0.000558

**Published:** 2021-04-13

**Authors:** Carla Perez-Mon, Weihong Qi, Surendra Vikram, Aline Frossard, Thulani Makhalanyane, Don Cowan, Beat Frey

**Affiliations:** ^1^​ Forest Soils and Biogeochemistry, Swiss Federal Institute for Forest, Snow and Landscape Research WSL, Birmensdorf, Switzerland; ^2^​ Functional Genomics Center of the University of Zurich and the ETH Zurich, Zurich, Switzerland; ^3^​ Centre for Microbial Ecology and Genomics, Department of Biochemistry, Genetics and Microbiology, University of Pretoria, Pretoria, South Africa

**Keywords:** alpine, microbial communities, metagenomics, permafrost, soil, warming

## Abstract

The warming-induced thawing of permafrost promotes microbial activity, often resulting in enhanced greenhouse gas emissions. The ability of permafrost microorganisms to survive the *in situ* sub-zero temperatures, their energetic strategies and their metabolic versatility in using soil organic materials determine their growth and functionality upon thawing. Hence, functional characterization of the permafrost microbiome, particularly in the underexplored mid-latitudinal alpine regions, is a crucial first step in predicting its responses to the changing climate, and the consequences for soil–climate feedbacks. In this study, for the first time, the functional potential and metabolic capabilities of a temperate mountain permafrost microbiome from central Europe has been analysed using shotgun metagenomics. Permafrost and active layers from the summit of Muot da Barba Peider (MBP) [Swiss Alps, 2979 m above sea level (a.s.l.)] revealed a strikingly high functional diversity in the permafrost (north-facing soils at a depth of 160 cm). Permafrost metagenomes were enriched in stress-response genes (e.g. cold-shock genes, chaperones), as well as in genes involved in cell defence and competition (e.g. antiviral proteins, antibiotics, motility, nutrient-uptake ABC transporters), compared with active-layer metagenomes. Permafrost also showed a higher potential for the synthesis of carbohydrate-active enzymes, and an overrepresentation of genes involved in fermentation, carbon fixation, denitrification and nitrogen reduction reactions. Collectively, these findings demonstrate the potential capabilities of permafrost microorganisms to thrive in cold and oligotrophic conditions, and highlight their metabolic versatility in carbon and nitrogen cycling. Our study provides a first insight into the high functional gene diversity of the central European mountain permafrost microbiome. Our findings extend our understanding of the microbial ecology of permafrost and represent a baseline for future investigations comparing the functional profiles of permafrost microbial communities at different latitudes.

## Data Summary

Nine supplementary tables and six supplementary figures are available with the online version of this article. The R scripts used for the statistical analyses are available at https://github.com/carlaperezmon/PerezMon_et_al_2020.

**Impact Statement**

Functional characterization of the permafrost microbiome in polar and alpine regions is a crucial first step in predicting the microbial responses of the soils to accelerated warming, and the consequences for the soil–climate feedbacks. Here, we applied shotgun DNA metagenomics to reveal, to the best of our knowledge for the first time, the functional gene diversity, survival strategies and metabolic potential for C and N cycling of a temperate mountain permafrost microbiome from central Europe. Our study, conducted at a long-term permafrost monitoring site in the Swiss Alps, the mountain summit of Muot da Barba Peider (MBP) [2979 m above sea level (a.s.l.)], used an elegant design on two aspects of a mountain ridge representing two distinct habitats. Collectively, our findings highlight the high functional diversity and spatial variability of the alpine permafrost microbiome. They represent a baseline for future investigations comparing the functional signatures of permafrost microbial communities at different latitudes.

## Introduction

Global warming is causing extensive thawing of permafrost soils, distributed in the Arctic, Antarctic and mid-latitudinal alpine regions [1–3]. Permafrost thawing increases microbial activity, often leading to enhanced soil greenhouse gas emissions [4–10]. Permafrost in polar and alpine regions represents different terrestrial cryoenvironments [7, 11] and harbours diverse microbial communities [12–14], whose carbon (C) and nitrogen (N) cycling functions, energetic metabolisms and survival are still not well understood [6, 9, 15, 16]. The ability of permafrost microorganisms to withstand the persistent sub-zero temperatures and their metabolic capabilities together determine their capacity for growth and functionality upon thawing [6, 9, 15, 17]. Hence, functional characterization of the permafrost microbiome at different latitudes is a crucial first step in predicting its responses to the changing climate, and the consequences for soil–climate feedbacks.

Functional information about the permafrost microbiome is mostly restricted to Arctic cryoenvironments [6, 9, 16, 18]. Arctic permafrost is usually overlaid by active layers with a depth of <1 m, and it exhibits temperatures ranging from 0 to below −10 °C [3, 7, 11]. The generally high moisture content of Arctic soils lead to suboxic conditions at the permafrost table [11]. Suboxia and the sub-zero temperatures promote the accumulation of poorly decomposed detritus in the permafrost, resulting in high soil organic carbon content (i.e. global SOC stock of 977 Pg) [7, 11]. In accordance with the suboxic conditions, meta-omic studies have shown that Arctic permafrost contains more genes related to anaerobic respiratory and fermentative metabolism than the overlying active layers [9, 19–22]. Permafrost microbiomes also feature a high abundance of carbohydrate-active enzymes (CAZys), which are involved in the decomposition of organic polymers [19, 21, 23, 24]. Moreover, permafrost metagenomes are enriched in genes involved in stress responses (e.g. cold-shock genes and chaperones), DNA repair, cell defence (e.g. antiviral proteins) and competition (e.g. antibiotics, motility) [15, 16, 21, 25–28].

Microbial studies in alpine permafrost regions are mainly in topsoils (i.e. at a depth of 5–10 cm) and they often lack functional information. Whereas the soil microbiome of the Tibetan mountains has been recurrently investigated, other mountain regions, e.g. in central Europe, remain underexplored [7]. Mid-latitudinal alpine permafrost is usually located on poorly vegetated steep slopes of mountains above 2500 m above sea level (a.s.l.), and dominated by coarse sediments that favour thermal conduction and the leaching of water and organic materials due to enhanced percolation [7, 10, 29]. In comparison to permafrost in polar regions, alpine permafrost is relatively warm (0 to −2 °C), and often found at soil depths >1 m. Furthermore, unlike the Arctic permafrost but similar to the Antarctic soils, alpine permafrost is well drained and depleted in organic materials (i.e. global SOC stock of 66 Pg) [7, 11].

GeoChip microarray surveys along altitudinal gradients in the Tibetan Plateau (3200 to 3800 m a.s.l.) indicated a higher abundance of cold-stress, carbon-fixation and denitrification genes in permafrost-like topsoils at higher altitudes [30, 31], which was associated with decreasing soil temperatures and organic C and N. Reduced inputs of labile C and N from fresh plant residues with increasing soil depths [32] and the preservation of poorly decomposed polymeric substrates at sub-zero temperatures [24] might contribute to habitat differences between the active layers and the permafrost. The abiotic differences between these soils might result in soil microbiota of distinct taxonomic composition [14] and metabolic features, including a greater genetic potential for the utilization of biopolymers in the permafrost. Furthermore, the large spatial and microenvironmental variability in alpine soils, owing to the extreme climatic heterogeneity of mountain regions [33–35], might be reflected in soil microbial communities with a particularly high diversity of functional genes.

Here, we analysed the microbial functional diversity and metabolic potential of an alpine permafrost soil and active layers from central Europe, using shotgun metagenomics. We compared soils at the same depths on two slopes from a well-characterized permafrost site in the Swiss Alps, the mountain summit of Muot da Barba Peider (MBP) (2979 m a.s.l.) [14, 36, 37]. Permafrost on MBP is found in the north-facing slope below a depth of 150 cm [14, 36, 37] and has an estimated age of 12 kiloyears, whereas soils on the south-facing slope at this depth are part of the active layer [14, 36, 37]. Like in other mountain systems in the Northern hemisphere, the south-facing topsoils of MBP exhibit higher temperatures and water-holding capacity than the north-facing topsoils, and a greater content of C and N, which together favour microbial life and the growth of plants [14]. A previous amplicon sequencing study conducted on MPB revealed that the soil microbial communities at different depths of the north- and south-facing slopes exhibit distinct diversities and structures [14]. In particular, permafrost soils in the north-facing slope were found to harbour a high proportion of metabolically versatile psychrophilic fungi and potentially parasitic bacterial taxa [14]. Our current study complements this earlier work and uses the same experimental design to analyse the functional gene profiles of the permafrost and the active layers. We first compared the metagenomes of north-facing permafrost soils collected at a depth of 160 cm (N160) with the metagenomes of north-facing active-layer soils collected at a depth of 10 cm (N10). We further explored the metabolic potential of the metagenomes in the south-facing active layers (S10 and S160) in comparison with the north-facing soils (N10 and N160). We hypothesized that, compared with the active layers, MBP permafrost (N160): (1) exhibits a distinct functional gene diversity and structure, (2) has a higher proportion of cold-stress genes and genes related to cell defence and competition, and (3) shows distinct C- and N-cycling genes, including a higher abundance of CAZys. We also hypothesize that (4) south-facing soils exhibit a higher functional diversity than north-facing soils.

## Methods

### Study site and sample collection

The study was conducted on the summit of MBP (N 46.49634 E 9.93145, 2979 m a.s.l.), located in the upper Engadine valley in eastern Switzerland (Fig. S1 available in the online version of this article). This site is part of the long-term monitoring of permafrost in the Swiss Alps [37]. Soil temperatures measured at a depth of 5 cm near the summit during 3 consecutive years (2016 to 2019), showed mean annual values of −2 °C (ranging from −14–21°C) in the north-facing slope and 1 °C (ranging from −8–24°C) in the south-facing slope. Mean annual precipitation in the region is 1500 mm [14]. The ground is covered with snow from October–November until May–June [38, 39]. The soil around the summit of MBP consists of coarse-grained materials in the uppermost 200 cm and finer-grained materials at greater depths [36]. The bedrock is found at 340–500 cm depth and consists of gneiss from the upper Austroalpine Languard nappe. Permafrost exists at 150 cm depth in the north-facing slope of the summit, whereas soils on the south-facing slope at this depth are part of the active layer [36, 37]. Soils are acidic and depleted in C (<1 % DW) and N (<0.1 % DW), especially in the north-facing slope (Table S1). Vegetation is scarce, and mostly occurs in the south-facing slope, with sparse observations of the taxa *Poa*, *Cerastium* and *Jacobea* spp. [14].

Soil samples were collected as described in detail previously [14]. Briefly, near the summit of MBP, six soil profiles were excavated with shovels down to a depth of 160 cm. Three profiles were excavated in both the north-facing and the south-facing slopes. The distance between the two sites over the mountain ridge was approximately 50 m. The external layer of the profiles, exposed to air, was removed with sterilized (70 % ethanol solution) spatulas, to eliminate debris and to prevent cross-contamination from upper soil layers. Bulk soil samples were collected from the pristine portion of the profiles at depths of 10 and 160 cm (2 slopes×2 soil depths×3 soil profiles=12 samples in total), using freshly sterilized spatulas. The collected samples (five subsamples of ≥100 g from each depth and profile) were homogenized in autoclaved bags, and roots were removed when present. Samples were transported in dry ice to the laboratory facilities, where they were immediately stored at −80 °C.

### Soil DNA extraction

Total DNA was extracted from 20 g of stored soil using a combination of the DNeasy PowerMax Soil kit and the DNeasy PowerSoil kit (Qiagen, Hilden, Germany). The soils were weighed into PowerMax Bead Tubes and the DNA extraction steps for (1) soil homogenization and cell lysis, and (2) removal of non-DNA materials from the lysates were completed using the PowerMax kit. The lysates were loaded into the spin columns of the PowerSoil kit, where (3) the DNA molecules bound into the silica membrane. The last extraction steps for (4) the washing and elution of the bounded DNA (in 100 µl of elution buffer) were performed using the PowerSoil kit.

All DNA extraction steps for the two kits were conducted according to the manufacturer’s instructions. To enhance cell lysis, an extra step was added to the PowerMax kit procedures. After weighing the soils into the PowerMax Bead Tubes and adding the PowerBead and the C1-SDS solutions, the tubes were vortexed (Vortex-genie 2, Scientific Industries, Inc., Bohemia, NY) for 15 min at a maximum speed of 3200 r.p.m. Then, the tubes were heated for 15 min at 65 °C in a shaking water bath. The combination of the two extraction kits complemented with the additional heating step yielded enough DNA (>0.1 ng µl^−1^) for sequencing. To eliminate foreign DNA and prevent microbial contaminations, working bench surfaces and non-autoclavable materials were cleaned with 5 % sodium hypochlorite and 70 % ethanol solutions, prior to the extractions. Negative controls (extraction buffer without soil) were included in the DNA extractions.

### Shotgun sequencing

Library preparation using the NEB Next ultra DNA Prep kit (Illumina, Inc., San Diego, CA) and shotgun sequencing of the eluted DNA samples were performed at the Genome Quebec Innovation Centre at McGill University (Montreal, Canada), using the HiSeq 2500 system (2×125 bp; Illumina, Inc.). The 12 metagenomes were from the 4 soil habitats (each with 3 replicates): active-layer soils collected at a depth of 10 cm in the north-facing (N10) and south-facing (S10) slopes, and soils collected at a depth of 160 cm in the north-facing (N160; permafrost) and south-facing (S160; active layer) slopes.

### Assembly and functional annotation of assembled contigs

Preprocessing of metagenomic reads, assembly of reads into contigs, contig binning, and functional and phylogenetic annotation of contigs and bins were achieved using a customized pipeline. Briefly, raw reads were quality checked using FastQC (https://www.bioinformatics.babraham.ac.uk/projects/fastqc/). They were quality filtered and trimmed (i.e. preprocessed reads) using Trimmomatic v0.36 (Q=20, minimum read length=40) [40]. Preprocessed read pairs and singletons were assembled into contigs (>200 bp) by iteratively building de Bruijn graphs using *k*-mers of increasing size with the *de novo* assembler MEGAHIT v1.1.3 (–k-min 27 –k-step 10) [41].

Protein-coding sequences contained in the assembled contigs were predicted with MetaGeneMark v3.38 [42]. To uncover the potential metabolic capabilities of the soil metagenomes, protein-coding genes were assigned to functions (i.e. functional genes). About 50 % of the predicted genes (4 706 835) were assigned to general metabolic and cellular functions through EggNOG v4.5, which classifies the genes to clusters of orthologous groups (COGs) of proteins and organizes the COGs into general functional categories [43]. Annotation to EggNOG v4.5 was performed using the eggnog-mapper v1.0.3 with DIAMOND search mode against all protein sequences [44]. The annotations obtained with EggNOG were compared with COG annotations obtained through the curated MD5nr database [45], in which 0.2 % of the genes (16 245) were assigned to functions. About 1 % (98 331) of the protein-coding genes were assigned to CAZys using the CAZy database (July 2017 release) [46]. Approximately 0.2 % of the genes (15 326) were assigned to N-cycling families using the NCycDB database. Annotations against the MD5nr, CAZy and NCycDB [47] databases were performed using SWORD v1.0.3 [48] (−v 10^−5^) [49].

### Abundance quantification of protein-coding genes

Preprocessed read pairs from each of the samples were mapped to the assembled contigs, using BWA Aligner v0.7.15 (bwa-mem) [50]. Mapping of the reads to the assembled protein-coding gene sequences to obtain gene abundances was done using the function *featureCounts* from the package Subread v1.5.1 (-minOverlap 10, Q=10, -primary) [51].

### Metagenome binning and phylogenetic annotation of genome bins

Contigs were binned with Metabat v2.12.1 [52], which iteratively clusters contigs into bins, based on similarities in abundance and tetranucleotide composition between pairs of contigs. Contig abundance was calculated with the *-jgi_summarize_bam_contig_depths* function, based on the bwa mem mapping of the preprocessed read pairs described in the previous section. Only contigs larger than 1500 bp and with a read depth >1 were included in the binning. Bins were assessed for quality (i.e. completion, contamination and strain heterogeneity) with CheckM v1.0.11 [53]. Bins with >90 % completion and <5 % contamination, presumably high-quality draft metagenome-assembled genomes [54], were classified phylogenetically using the same program. The *-ssu_finder* function in CheckM was applied to identify SSU rRNA sequences in the contigs within the bins (16S for prokaryotes and 18S for eukaryotes). These sequences were annotated taxonomically via the sina online aligner v1.2.11 [55] against the silva database (release 132) [56].

### Data analyses

Statistical analyses were completed using the open-source software R v3.6.0 [57] and graphical representations of results were created with the R package *ggplot2* [58], unless specified otherwise. A significance level (*P*) of 0.05 was applied in all analyses. Protein-coding genes for which the sum of the reads over all soil samples was <10 were excluded from the analyses to lower the false discovery rate caused by stochasticity between samples.

Differences in protein-coding gene richness and Shannon diversity between the soil habitats were assessed with Welch’s *t*-tests followed by Games–Howell post-hoc tests, after verifying that residuals were normally distributed but that variance of the response variable differed between the groups. Differences in functional structure between the soils were assessed by computing Bray–Curtis dissimilarity matrices based on the read abundance of the protein-coding genes normalized to transcripts per million (t.p.m.) [59]. Bray–Curtis dissimilarities between samples were visualized with principal coordinate analyses (PCoAs) (*vegan* R package [60]). The statistical significance of observed differences was assessed with permutational analyses of variance (PERMANOVA, 10^5^ permutations, Monte Carlo approximated *P* value) using PRIMER v7 [61]. Multivariate homogeneity of group dispersions was checked prior to the PERMANOVAs, also with PRIMER v7, to ensure that detected significant differences were associated with the tested factors and not with differences in the within-group variabilities.

To evaluate the dissimilarities in metabolic capabilities between the four soil habitats, we identified the functional genes – annotated against EggNOG, MD5nr, CAZy and NCycDB databases – that were differentially abundant between the soils using pairwise DESeq2 analyses [62]. For all pairwise comparisons median-of-ratio normalization was applied to account for differences in sequencing depth among samples. *P* values were adjusted for multiple testing using the Benjamini–Hochberg method with a false discovery rate threshold of 5 %.

## Results

### Protein-coding genes diversity

Whole-metagenome sequencing of the 12 soil metagenomes (N10, N160, S10, S160) generated an average of 40 to 45 million high-quality reads per soil (Table 1). Megahit assembly of reads into contigs produced a total of 6 627 330 contigs of 713 bp on average, ranging from 200 to 939 240 bp and with a N50 value of 761 bp. A total of 9 837 348 different protein-coding genes were predicted from the assembled contigs. Gene quantification of the contigs associated with the different soil habitats revealed that soil metagenomes contained on average 51–58 % protein-coding sequences, except for the S160 soils, where values reached up to 77 % (Table 1).

**Table 1.** Total number of sequences and percentage of protein-coding genes and contigs assigned to taxa for the soils of different origins. Mean±sd (*n*=3)

	N10			N160			S10			S160		
Raw reads (10^6^)	41	±	4	46	±	8	46	±	3	46	±	4
HQ reads (10^6^)	39	±	3	45	±	8	45	±	3	45	±	3
Reads mapped to contigs (%)	54	±	7	58	±	1	54	±	7	80	±	2
Reads mapped to CDS genes* (%)	51	±	8	58	±	1	55	±	6	77	±	0
Taxonomically assigned contigs† (%)	99.6	±	0.1	99.3	±	0.3	99.8	±	0.2	99.7	±	0.1
Archaea (%)	0.6	±	0.4	0.3	±	0.1	0.4	±	0.1	0.8	±	0.2
Bacteria (%)	87.1	±	8.2	97.7	±	0.6	93.9	±	6.7	98.4	±	0.6
Eukarya (%)	11.9	±	8.6	1.3	±	0.4	5.4	±	6.6	0.5	±	0.6

*CDS genes, protein-coding genes.

†From a total of 823 contigs >1500 bp in which SSU regions >300 bp were identified.

N, north-facing; S, south-facing; 160, at a depth of 160 cm; 10, at a depth of 10 cm.

**Fig. 1.**
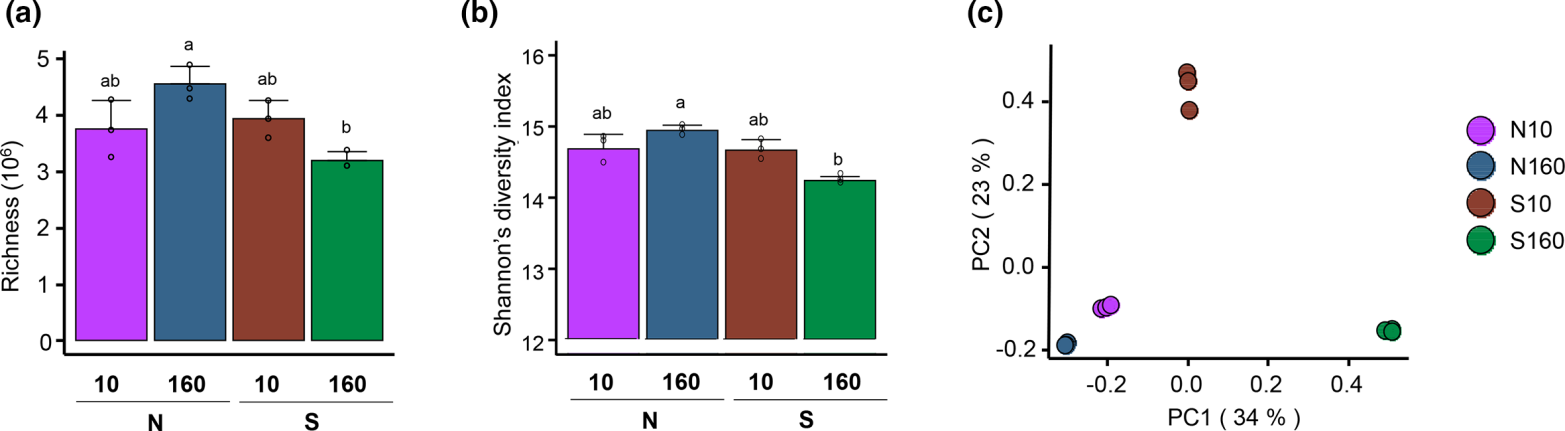
Functional diversity and structure of protein-coding genes for the different soil habitats. (**a**) Number of different protein-coding genes (i.e. richness). (**b**) Shannon’s diversity index based on read abundance normalized to transcripts per million (t.p.m.). Barplots depict the mean±sd of the diversity values for the soil groups, whereas open dots represent the diversity values of the individual soils. (**c**) Principal coordinate analyses (PCoAs) computed on Bray–Curtis dissimilarities based on t.p.m. normalized abundances of the protein-coding genes. Only protein-coding genes for which the sum of the reads over all soil samples was ≥10 are included. N, north-facing; S, south-facing; 10, at a depth of 10 cm; 160, at a depth of 160 cm.

Soil metagenomes differed in their functional diversity and structure (Figs 1 and S2). The richness and Shannon’s diversity of the predicted protein-coding genes were highest for the N160 soils and lowest for the S160 soils (Fig. 1). N160 (permafrost) soils significantly (*P* <0.05; PERMANOVA) differed from the S160 soils (active layer) in their protein-coding gene structures (Fig. 1, Table S2). Interestingly, the N10 and N160 soils clustered closely together (*P* > 0.05), indicating an overall similar composition of protein-coding genes.

### Overall metabolic capabilities of the soil metagenomes

**Fig. 2.**
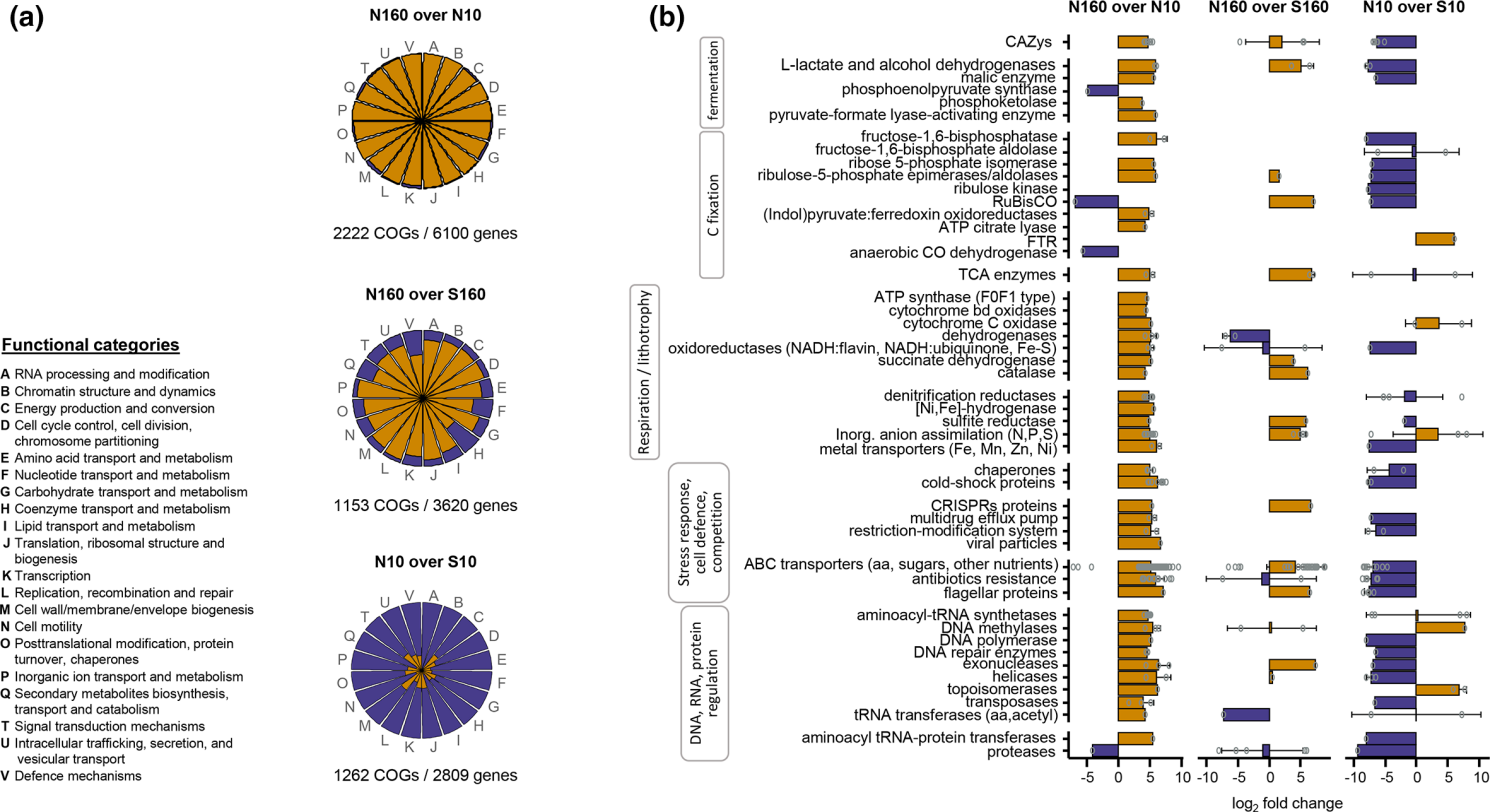
Overall functional differences between the different soil habitats. (**a**) Relative abundance of overrepresented COGs (orange) and underrepresented COGs (violet) distributed across COG functional categories, for the comparisons between north-facing permafrost and active-layer soils (N160 over N10), between north-facing permafrost and south-facing active-layer soils at a depth of 160 cm (N160 over S160), and between north-facing and south-facing active-layer soils at a depth of 10 cm (N10 over S10). The log_2_ fold change (LFC) value ‘N160 over N10’ is the log_2_ of (gene abundance of N160/gene abundance of N10). The same stands for ‘N160 over S160’ and ‘N10 over S10’. For each pairwise comparison, highly differentially abundant genes (*P* <0.01) among the 80 % most abundant genes assigned to COGs were selected. The represented COGs are those in which all selected genes were overrepresented (LFC >0) or underrepresented (LFC <0). Values below the pie charts indicate the total number of different COGs and genes included in the representations. (**b**) LFC in selected differentially abundant COGs assigned to proteins associated with C cycling and relevant metabolic and cellular processes for the compared soil habitats. COGs were assigned to proteins using the EggNOG database and annotations were curated with the non-redundant MD5nr database. Barplots depict the mean±sd of the LFC values for the protein groups, whereas open dots represent the LFC values of the individual COG genes. A list of all selected genes with their relative abundance, their COG classification and the assigned functions is provided in Table S5. RuBisCO, ribulose-1,5-bisphosphate carboxylase; FTR, formylmethanofuran:tetrahydromethanopterin formyltransferase.

DESeq2 analyses of protein-coding genes assigned to functions (i.e. functional genes) enabled us to uncover differences in potential metabolic capabilities between the soil metagenomes. N160 (permafrost) metagenomes showed a higher diversity (richness and Shannon’s diversity index, Table S3, Fig. S3) and a higher abundance of genes (log_2_ fold change >0) assigned to different COGs (EggNOG and MD5nr databases) than the N10 and the S160 metagenomes (Fig. 2a, Table S4). Differentially abundant COGs were distributed across all functional categories, suggesting that differences in potential metabolic capabilities between the soil habitats comprise a broad range of intracellular and extracellular processes (Fig. 2, Table S5). Permafrost metagenomes contained a higher proportion of: genes coding for cold-shock proteins and chaperones, involved in stress responses; restriction nucleases and CRISPRs proteins involved in cell defence; and antibiotics, flagellar proteins and ABC transporters related to competition (Fig. 2b, Table S5). Genes involved in chromatin remodelling and transcription regulation processes, including DNA repair, were also overrepresented in the permafrost soils (Fig. 2b), whereas only a few sporulation genes were detected across all the soils (Table S5). Proteins involved in anaerobic and aerobic respiratory metabolism were also overrepresented in the permafrost metagenomes. These included transporters of metals and inorganic anions, N and S reductases, [NiFe]-hydrogenases, and catalases and oxidoreductases from the aerobic respiratory chain (Fig. 2b, Table S5).

Permafrost soils showed a higher abundance of genes involved in fermentative processes (e.g. those coding for phosphoketolase, and alcohol and l-lactate dehydrogenases) than the N10 soils and, to a lesser extent, the S160 soils (Fig. 2b, Table S5). Genes coding for key enzymes involved in anaerobic C fixation (pyruvate : ferredoxin oxidoreductase and citrate lyases) were also enriched in the permafrost metagenomes relative to those from the active layers.

Differences in COG diversity (Table S3, Fig. S3) and abundance were apparent between the N10 and S10 soils (Fig. 2, Tables S4 and S5). Overall, S10 soils exhibited a higher abundance of COGs involved in all analysed cellular and extracellular processes than the north-facing soils. S10 vs S160 comparisons also showed a higher abundance of COGs in the S10 soils (data not shown).

### Carbohydrate-active enzyme genes

**Fig. 3.**
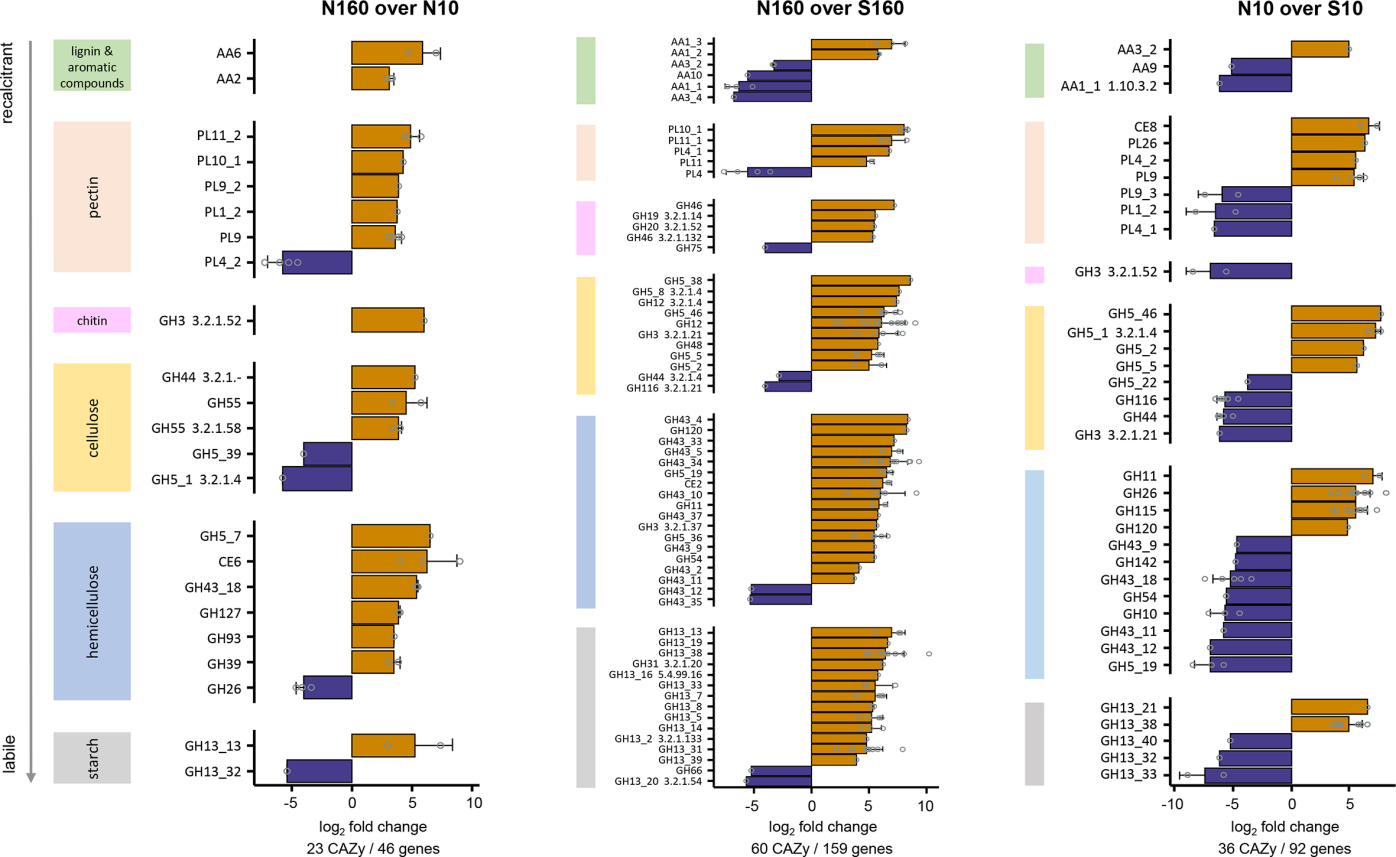
Differences in CAZy abundance between the different soil habitats. Pairwise comparisons were conducted between north-facing permafrost and active-layer soils (N160 over N10), between north-facing permafrost and south-facing active-layer soils at a depth of 160 cm (N160 over S160), and between north-facing and south-facing active-layer soils at a depth of 10 cm (N10 over S10). The log_2_ fold change (LFC) value ‘N160 over N10’ is the log_2_ of (gene abundance of N160/gene abundance of N10). The same stands for ‘N160 over S160’ and ‘N10 over S10’. For each pairwise comparison, differentially abundant genes (*P* <0.05) that were among the 80 % most abundant genes assigned to CAZys were selected. The represented CAZys are those in which all selected genes were overrepresented (LFC >0) or underrepresented (LFC <0). CAZy families and proteins were linked to specific substrates based on their decomposing activities. Only families associated with a unique decomposing activity or mostly with a specific decomposing activity (the same activity was described for at least 50% of all characterized enzymes in families with a total of ≤3 enzymes, http://www.cazy.org/ browser) are represented. CBM families are not shown. Barplots depict the mean±sd of the LFC values for the CAZy families, whereas open dots represent the LFC values of the individual genes. Values below the bar graphs indicate the total number of CAZys and genes included in the representation. A list of all selected genes with their relative abundance, CAZy classification and associated enzymatic activity is provided in Table S6.

The most abundant CAZy genes (CAZy database) across all MBP soils coded for glycosyl transferases, followed by glycoside hydrolases, carbohydrate-binding modules, carbohydrate esterases, auxiliary activity enzymes and polysaccharide lyases (Table S3). N160 soils had a higher diversity (Table S3, Fig. S4) and a higher abundance of genes coding for different CAZys (i.e. families and individual proteins) than the N10 and S160 soils (Fig. 3, Tables S4 and S6). Overrepresented CAZys in the permafrost soils included peroxidases (AA1 to AA10), pectate lyases (PL families) and cellulases (GH families), which are involved in the decomposition of complex plant components (i.e. lignocellulose and pectin) (Fig. 3, Table S6). Permafrost metagenomes were also enriched in CAZys involved in the use of labile substrates (i.e. starch) (Fig. 3). These included genes coding for pullulanases, α-amylases and α-glucosidases within the GH13 families (Fig. 3, Table S6). CAZys involved in chitin depolymerization (e.g. β-N-acetylhexosaminidases) were also overrepresented in the permafrost.

The diversity of CAZy genes was similar in the active layers from the north- and south-facing slopes (N10 over S10) (Table S3, Fig. S4). The S10 soils showed a higher abundance of CAZys involved in the depolymerization of hemicellulose (e.g. xylanases) and labile starch (e.g. α-glucosidases) than the N10 soils (Fig. 3, Table S6).

### N-cycling genes

**Fig. 4.**
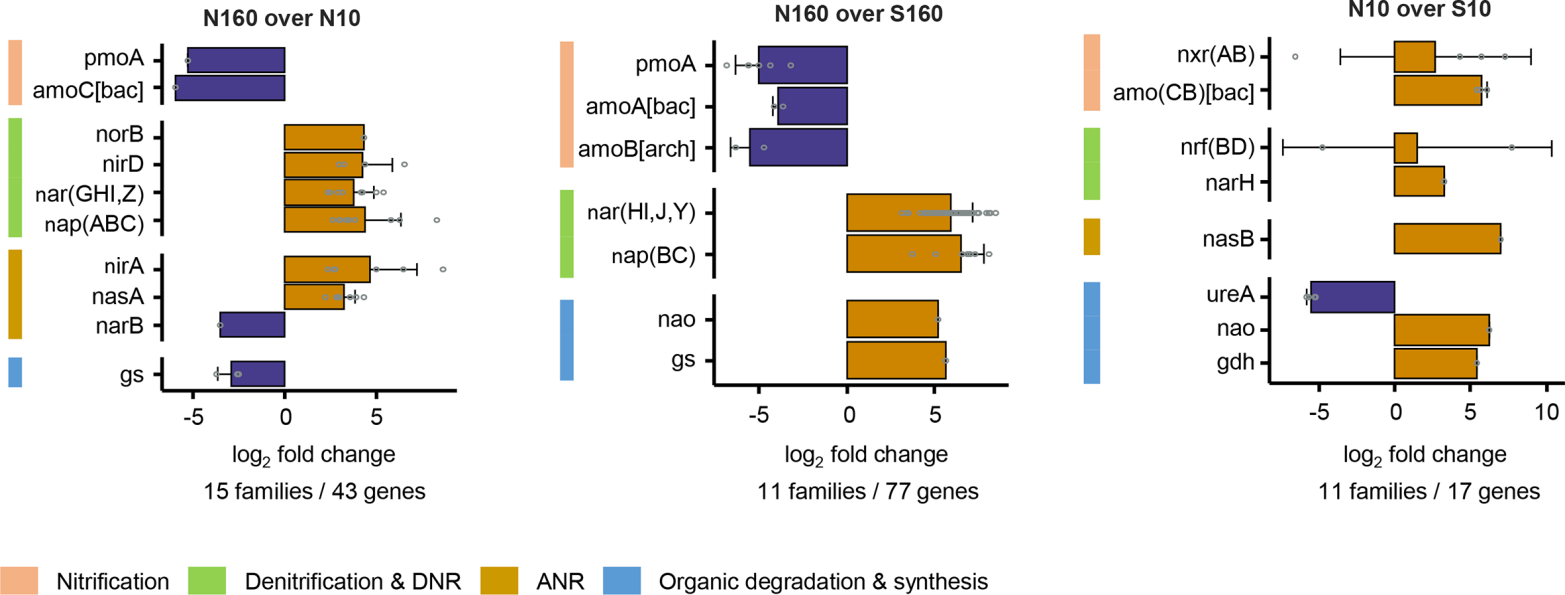
Differences in abundance of N-cycling families between the different soil habitats. Pairwise comparisons were conducted between north-facing permafrost and active-layer soils (N160 over N10), between north-facing permafrost and south-facing active-layer soils at a depth of 160 cm (N160 over S160), and between north-facing and south-facing active-layer soils at a depth of 10 cm (N10 over S10). The log_2_ fold change (LFC) value ‘N160 over N10’ is the log_2_ of (gene abundance of N160/gene abundance of N10). The same stands for ‘N160 over S160’ and ‘N10 over S10’. For each pairwise comparison, differentially abundant genes (*P* <0.05) that were among the 80 % most abundant genes assigned to N-cycling families were selected. The represented N-cycling families are those in which all selected genes were overrepresented (LFC >0) or underrepresented (LFC <0). Barplots depict the mean±sd of the LFC values for the N-cycling families, whereas open dots represent the LFC values of the individual genes. Values below the bar graphs indicate the total number of N-cycling families and genes included in the representations. amo (ABC), ammonia monooxygenase; gdh, glutamate dehydrogenase; gs, glutamate synthase; nao, nitroalkane oxidase; nap (ABC), nitrate reductase; nar(GHIJYZ), nitrate reductase; nas (AB), assimilatory nitrate reductase; nir (AD), nitrite reductase; nrf (BD), nitrite reductase; nxr (AB), nitrate reductase; pmoA, particulate methane monooxygenase; ureA, urease; bac, Bacteria; arch, Archaea. A list of all selected genes with their relative abundance and their classification to N-cycling families and processes is provided in Table S7.

N160 soils exhibited a higher diversity (Table S3, Fig. S5) and a higher abundance of N-cycling families (NCycDB database) involved in denitrification, dissimilatory nitrogen reduction (DNR) (e.g. *nor*, *nir* and *nar* genes) and assimilatory nitrogen reduction (ANR) (e.g. *nas* genes) than the N10 and S160 soils (Fig. 4, Table S7). Conversely, nitrification genes (e.g. *amo*) were less abundant in the permafrost than in the other soils. Comparisons between topsoils (N10 over S10) revealed a higher abundance of N-cycling families involved in both nitrification and denitrification in the S10 soils, including genes for the anabolic and catabolic processing of organic N (i.e. *ure*, *nao* and *gdh*) (Fig. 4, Table S7).

### Taxonomic analysis of the soil communities and functional assignment to taxa

A total of 823 contigs, larger than 1500 bp and with SSU rRNA (small subunit ribosomal ribonucleic acid) regions larger than 300 bp, were identified. Taxonomic annotation of these SSU rRNA-harbouring contigs revealed that the different soil habitats were dominated by Bacteria (Table 1). Archaea represented <1 % of the soil microbiota. Eukaryotic contigs were more abundant in the topsoils (S10 and N10), where abundances ranged from 5–12 % of the total abundance of all contigs assigned to taxa (Table 1). Eukaryotes mostly consisted of fungi, but a large variety of sequences belonging to plants, protists and, to a lesser extent, invertebrates were also detected across the different soil habitats (Fig. S6).

**Fig. 5.**
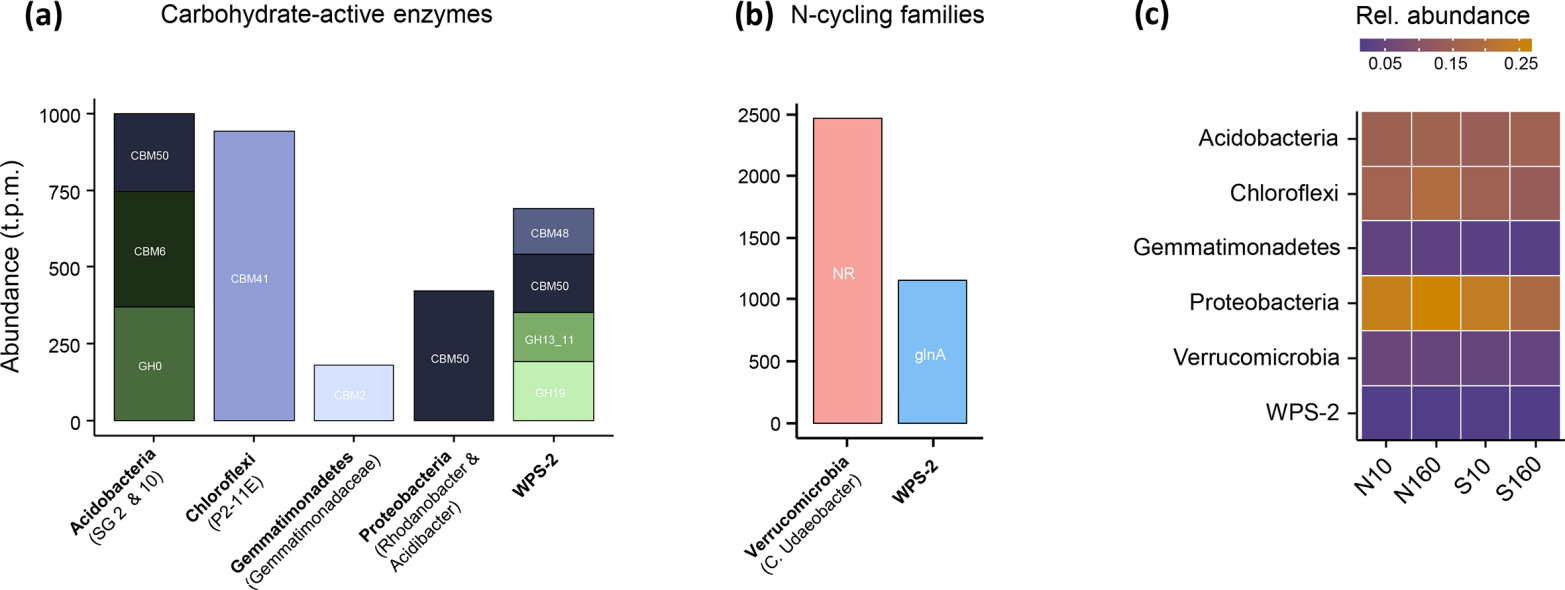
Bacterial phyla containing CAZys (**a**) and N-cycling families (**b**), and their abundances (**c**), across the different soil habitats. Contigs larger than 1500 bp were selected and bacterial taxa were identified by aligning 16S rRNA genomic regions (>300 bp) against the silva database. Rel. abundance represents the mean proportion of the taxa in the different soil habitats relative to the total read depth of all bacterial contigs. N, north-facing; S, south-facing; 160, at a depth of 160 cm; 10, at a depth of 10 cm.

The most abundant bacterial phyla were Proteobacteria, Chloroflexi, Acidobacteria, Actinobacteria, Verrucomicrobia, Gemmatimonadetes, Planctomycetes, Patescibacteria, Bacteroidetes and WPS-2 (i.e. Eremiobacterota), with abundances higher than 1 % of the total abundance of all bacterial contigs (Table S8, Fig. S6). Contigs assigned to these phyla were found to contain CAZy genes coding for glycoside hydrolases (GHs) and carbohydrate-binding modules (CBMs), which govern the binding of the hydrolytic enzymes to their substrates (Fig. 5). Acidobacteria and WPS-2 contigs contained the highest proportion of CAZys. WPS-2 contigs also contained glutamine synthetase (GlnA) genes, involved in the metabolism of organic N (Fig. 5).

Binning of contigs produced 15 highly completed metagenome-assembled genomes (MAGs; >90 % completion, <5 % contamination). Most of these MAGs were phylogenetically classified to Acidobacteria and Proteobacteria (Table S9).

## Discussion

Functional profiling of permafrost microbial communities has largely focused on high-latitudinal soils, whereas the functional gene diversity of the oligotrophic, coarse-drained permafrost soils from mid-latitudinal alpine regions is poorly known. In this novel study, we applied shotgun DNA metagenomics to assess the metabolic potential of a temperate mountain permafrost microbiome from central Europe. Our study, conducted at the long-term permafrost monitoring site of Muot da Barba Peider (MBP) in the Swiss Alps, involved a functional gene comparison, not only between permafrost (160 cm depth) and the overlaying active layer (10 cm depth), but also between active-layer habitats with different expositions and therefore different mean annual temperatures. This latter comparison is not often possible in polar ecosystems.

In MBP, permafrost had the highest functional gene diversity among the soils considered, which corresponds to the high taxonomic diversity previously described at this site [14]. In agreement with our first hypothesis, MBP permafrost (N160) showed a functional structure that was distinct from that of the south-facing active-layer soils collected from the same depth (S160) and to a minor extent from the north-facing active layer (N10). Analysis of differentially abundant genes between the soil habitats revealed that, in line with our second hypothesis, permafrost was enriched in genes involved in stress responses, including cold-shock genes and chaperones, and those involved in cell defence, such as the antiviral CRISPRs proteins. Permafrost environments are dominated by the hostile-to-life conditions of constant freezing, desiccation and starvation [15]. In line with findings from Arctic and Antarctic environments [20, 23, 63, 64], the higher potential of the MBP permafrost microorganisms to synthesize cold-shock proteins and chaperones could indicate their ability to survive prolonged freezing, as these proteins ensure the integrity of the cell components and the continuing functioning of the cells under environmental stress [65].

In permafrost, microorganisms might thrive in small pockets of liquid water within the soil pores, which contain metabolic substrates [12, 23, 26, 27]. The cohabitation of the microbial cells in proximity would promote virulence [26], which, as underlined in previous permafrost investigations [15, 26], could be related to the higher proportion of cell defence genes within the MBP permafrost. Microbial groups might compete to colonize the habitable liquid pockets in the permafrost and profit from the limited bioavailable C and nutrient resources [27]. As described for Arctic soils, enhanced microbial competition could be associated to the overrepresentation of antibiotic-related genes (e.g. β-lactamases) in MBP permafrost, as well as motility proteins and ABC transporters associated with the microbial search and uptake of simple C and nutrient sources from the surrounding environment [27, 66].

Counterintuitively, relatively few sporulation genes were identified in the MBP soils. Instead, genes involved in chromatin remodelling, including DNA repair mechanisms, replication and biogenesis, characteristic of microorganisms in an active state [15, 67, 68], were overrepresented in the permafrost. Similar observations have been reported in Arctic permafrost [21, 26, 27], where bacterial growth at sub-zero temperatures has been demonstrated for isolated strains [67–69]. As previously suggested [21, 27, 70], and despite the elevated energetic costs, permafrost microorganisms might retain activity because the risk of DNA degradation over time might select against spore formation as a long-term survival strategy [21, 27, 70]. This is further supported by preliminary flow cytometry measurements in MBP soil samples, which indicate the existence of up to 40 % living microbial cells in the permafrost (live-dead staining, unpublished data). This notwithstanding, the low abundance of sporulation genes in the MBP soils could be partly due to a limited recovery of DNA from spore-forming microorganisms during the DNA extractions. Spore lysis often requires particularly strong mechanical (e.g. bead-beating at speeds >4 m s^−1^) and chemical treatments [71] that were not included in our DNA extraction method.

The higher functional diversity observed in the MBP permafrost soils compared with the active-layer soils is surprising. Microbial communities in Arctic permafrost have been found to be highly diverse, both taxonomically [9, 12, 20] and functionally [19–21, 23, 72–74]. However, the taxonomic and functional diversity values reported from Arctic soils are often higher in the active layers than in the permafrost [19, 20, 22, 23, 73, 74], likely linked to the retention of fresher and more diverse organic substrates in shallow and aerated soil layers [74]. The coarse-grained texture characteristic of alpine soils, such as the north-facing slopes of MPB, facilitates thermal conduction and the leaching of water and organic materials, as a result of enhanced percolation [10, 29]. The higher functional gene diversity in the MBP permafrost could be explained by the selective pressure imposed by freezing combined with the adaptation of the microorganisms inhabiting pockets of liquid water to different conditions of pH, redox and substrates [21, 23]. Alternatively, the higher diversity of genes detected in the permafrost might be attributed to the accumulation of genetic material over geological timescales, preserved by the sub-zero temperatures [75, 76].

In support of our third hypothesis, permafrost samples contained a higher diversity of CAZy genes than the active layers, coding for multiple glycosyl hydrolases (GHs), auxiliary enzymes and pectate lyases that together participate in the depolymerization of plant-derived recalcitrant (i.e. lignocellulosic and phenolic) compounds, labile polymers (i.e. starch) and microbial detritus (i.e. chitin). As in the Arctic, alpine permafrost microorganisms might preferentially utilize polymeric organic materials as primary sources of C, nutrients and energy [19, 27, 77]. Permafrost from Alaska [23, 24], Svalbard [21] and northern Sweden [19] has been shown to be enriched in CAZy genes. Likewise, Biolog assays applied to Siberian soils [73, 74] demonstrated the ability of the permafrost microorganisms to grow on complex biopolymers. In the mountain environments of the Tibetan Plateau, detailed characterization of the organic matter composition and microbial degradation patterns in permafrost and active layers revealed the existence of rich deposits of labile and recalcitrant C in the permafrost, both undergoing microbial degradation [10].

Interestingly, in MBP soils, CAZy genes coding for GH and carbohydrate-binding modules (CBM) were found to be associated with bacterial taxa, particularly Acidobacteria and WPS-2. This observation might suggest that these phyla are important contributors to C cycling in these soils [19]. Acidobacteria and WPS-2 are typically widespread in acidic soils, including those from alpine, Arctic and Antarctic regions [12–14, 78, 79], and have been shown, in the multi-omic investigation of permafrost soils from Stordalen Mire (northern Sweden), to be dominant C degraders [19]. WPS-2 (i.e. *Candidatus* Eremiobacterota) [80] is an uncultured phylum, originally identified in Antarctic soil metagenomes. There are a few draft genomes available from this clade [78, 80] where anoxygenic photosynthesis and chemosynthesis have been identified. Future analyses of the functional features of the highly completed MAGs recovered in the present study, which included numerous Acidobacteria, will facilitate insights into the ecological roles of alpine permafrost microorganisms and their metabolic interactions.

MBP permafrost also showed a higher abundance of fermentative and carbon-fixation genes than the active layers, indicating the potential use of simple sugars for carbon and energy gain, together with the use of carbon dioxide to support primary production. Furthermore, MBP permafrost was enriched in N-cycling genes involved in denitrification and anaerobic nitrate reduction, whereas genes involved in nitrification and organic N transformation were underrepresented in the permafrost compared with the active layers. GeoChip microarrays along altitudinal gradients (3200 to 3800 m a.s.l.) in the Tibetan Plateau showed an increase in genes for carbon fixation and denitrification at a soil depth of 5–10 cm at higher altitudes, which was correlated with a decrease in organic C and N content with increasing altitude [30, 31]. In Arctic permafrost, fermentative metabolism and denitrification (i.e. nitrate and nitrite reduction) have often been observed [9, 19–22, 81], although they have mostly been attributed to the anoxic niche conditions. Novel strains isolated from MBP have been reported to use a wide range of carbon sources and to assimilate nitrite and nitrate [82]. Further studies should include genome-guided insights into the versatile metabolic capabilities and process-based measurements (e.g. respiration, N_2_O) *in situ* or under controlled conditions to unveil the active members of the MBP permafrost microbiome.

Interestingly, the MBP permafrost showed a high proportion of genes involved in both aerobic and anaerobic respiratory metabolism (e.g. iron (III), sulphate reductases and [NiFe]-hydrogenases). This could be linked to the well-drained coarse sediments that would enable aeration and thereby support the co-occurrence of aerobic and anaerobic microorganisms in the permafrost [21, 23]. The detection of [NiFe]-hydrogenases in the MBP permafrost is noteworthy. [NiFe]-hydrogenases are involved in the utilization of hydrogen for energy acquisition [83]. Recent genomic studies indicated that hydrogen metabolism is widespread in prokaryotes [83], and might be predominant in oligotrophic Antarctic soils [80, 84, 85]. The possibility that this energy-acquisition strategy is also prevalent in the temperate mountain permafrost microbiome is worthy of further investigation. Overall, more functional metagenomic studies in both Antarctic and alpine permafrost are needed, for comprehensive functional comparisons between the microbial communities of these desert soil ecosystems.

In agreement with our fourth hypothesis, the south-facing topsoils (S10) of MBP showed a higher functional gene diversity than the north-facing active layers (N10), including a greater variety of C metabolic pathways and genes involved in the processing of organic N. We also found a higher abundance of CAZy genes, potentially involved in the depolymerization of hemicellulose and starch, in the S10 soils. The S10 soils have higher annual mean soil temperature, moisture and concentrations of C and N than the N10 soils, thereby representing a more favourable habitat for microbial life. The higher functional gene diversity of the MBP permafrost (N160) compared with the S160 soils might be linked to its particular habitat characteristics (i.e. frozen soils putatively seeded with liquid microniches of varying environmental conditions), and its high taxonomic diversity.

In the future, the environmental conditions of the north-facing soils may resemble the current conditions of south-facing soils to some extent, owing to the projected climate change-related temperature increases and denser vegetation cover [2, 86]. Incubation of MBP permafrost [87, 88] and permafrost-affected mineral soils [89–91] at elevated temperatures indicates an increase in the abundance of metabolically versatile microbial taxa. These taxonomic changes could be coupled to the increase in microbial C decomposition and organic N cycling, as suggested in genomic surveys of Arctic soils undergoing warming [26, 92–94]. Further research is needed to identify the active metabolisms of the MBP permafrost microbiota (e.g. through metatranscriptomics and metaproteomics) and its functional responses to warming.

## Conclusions

Our study on the functional characteristics of the MBP metagenomes underlines the remarkably high functional gene diversity of the temperate mountain permafrost microbiome, including a broad range of C- and N-cycling genes, and multiple survival and energetic metabolisms. Our findings indicate that permafrost in European alpine regions might comprise an important genetic reservoir, not only for the study of poorly characterized microbial metabolisms (e.g. H_2_ metabolism), but also for proteins of pharmaceutical interest (e.g. antibiotics and CRISPRs); hence, our results underscore the need to extend investigations to other temperate mountain permafrost soils. Our findings represent a baseline for future investigations comparing the functional profiles of permafrost microbial communities at different latitudes, which in turn will widen our understanding of the global permafrost microbiome.

## Supplementary Data

Supplementary material 1

Supplementary material 2
