# Supplementary material for: Shotgun metagenomics reveals distinct functional diversity and metabolic capabilities between 12 000-year-old permafrost and active layers on Muot da Barba Peider (Swiss Alps)
Source: Microb Genom. 2021 Apr 13;7(4):000558. doi: 10.1099/mgen.0.000558 (PMC8208683; doi:10.1099/mgen.0.000558)
Supplement: Supplementary material 1 [file mgen-7-0558-s001.pdf]

SUPPLEMENTARY INFORMATION

Shotgun metagenomics reveals distinct functional diversity and metabolic capabilities between 12 000 year old permafrost and active layers on *Muot da Barba Peider* (Swiss Alps)

Authors

Carla Perez-Mon[1*], Weihong Qi[2], Surendra Vikram[3], Aline Frossard[1], Thulani Makhalanyane[3], Don Cowan[3], Beat Frey[1*]

Affiliation

[1]Forest Soils and Biogeochemistry, Swiss Federal Institute for Forest, Snow and Landscape Research, Birmensdorf, Switzerland

[2]Functional Genomics Center of the University of Zurich and the ETH Zurich, Zurich, Switzerland

[3]Centre for Microbial Ecology and Genomics, Department of Biochemistry, Genetics and Microbiology, University of Pretoria, Pretoria, South Africa

∗**Corresponding authors**

Beat Frey, Swiss Federal Research Institute WSL, Zuercherstrasse 111, CH-8903 Birmensdorf, Switzerland. Tel: +41-44-739-25-41; E-mail: beat.frey@wsl.ch. Carla Perez-Mon, Swiss Federal Research Institute WSL, Zuercherstrasse 111, CH-8903 Birmensdorf, Switzerland, carla.perezmon@wsl.ch

Supplementary figures legends

Fig. S1. Geographic location of *Muot da Barba Peider* with an aerial view of the summit (a), and schematic representation of the soil sample collection (b). The blue fill dot represents the MBP mountain. The aerial photography of MBP was provided by Dr. Marcia Phillips. Pictures overlapping the aerial photography of MBP show the north-facing and south-facing sampling locations at the summit, accompanied with a close visualization of the topsoils. The solid line in (b) represents the permafrost table at a depth of 150 cm b.g.s. N: north-facing, S: south-facing, 10: at a depth of 10 cm, 160: at a depth of 160 cm.

Fig. S2. Functional structure of the different soil habitats for the genes assigned to functions. PCoAs computed on Bray-Curtis dissimilarities based on the abundance of selected genes for which the sum of the reads over all soil samples was ≥ 10. N: north-facing, S: south-facing, 10: at a depth of 10 cm, 160: at a depth of 160 cm.

Fig. S3. Shannon's diversity of COGs grouped into functional categories for the different soil habitats. Shannon's diversity indexes were calculated on read abundances normalized to transcripts per million (t.p.m.). The represented genes are those for which the sum of the reads over all soil samples was ≥ 10. Mean ± SD (n=3). N: north-facing, S: south-facing, 10: at a depth of 10 cm, 160: at a depth of 160 cm.

Fig. S4. Shannon's diversity of CAZys classes for the different soil habitats. Shannon's diversity indexes were calculated on read abundances normalized to transcripts per million (t.p.m.). The represented genes are those for which the sum of the reads over all soil samples was ≥ 10. Mean ± SD (n=3). N: north-facing, S: south-facing, 10: at a depth of 10 cm, 160: at a depth of 160 cm.

Fig. S5. Shannon's diversity of N-cycling processes for the different soil habitats. Shannon's diversity indexes were calculated on read abundances normalized to transcripts per million (t.p.m.). The represented genes are those for which the sum of the reads over all soil samples was ≥ 10. ANR: assimilatory nitrogen reduction, DNR: dissimilatory nitrogen reduction. Mean ± SD (n=3). N: north-facing, S: south-facing, 10: at a depth of 10 cm, 160: at a depth of 160 cm.

Fig. S6. Prokaryotic and eukaryotic composition of the different soil habitats. Taxonomic classification was performed on the contigs larger than 1500 bps, in which SSU regions (> 300 bp) could be identified. Contigs were annotated against the SILVA database. The represented bacterial taxa are those for which the depth of coverage was ≥ 2% of the total depth of coverage over all bacterial contigs assigned to taxa beyond the kingdom level. Taxa were classified to the deepest taxonomic level possible. N: north-facing, S: south-facing, 10: at a depth of 10 cm, 160: at a depth of 160 cm.

Fig. S1

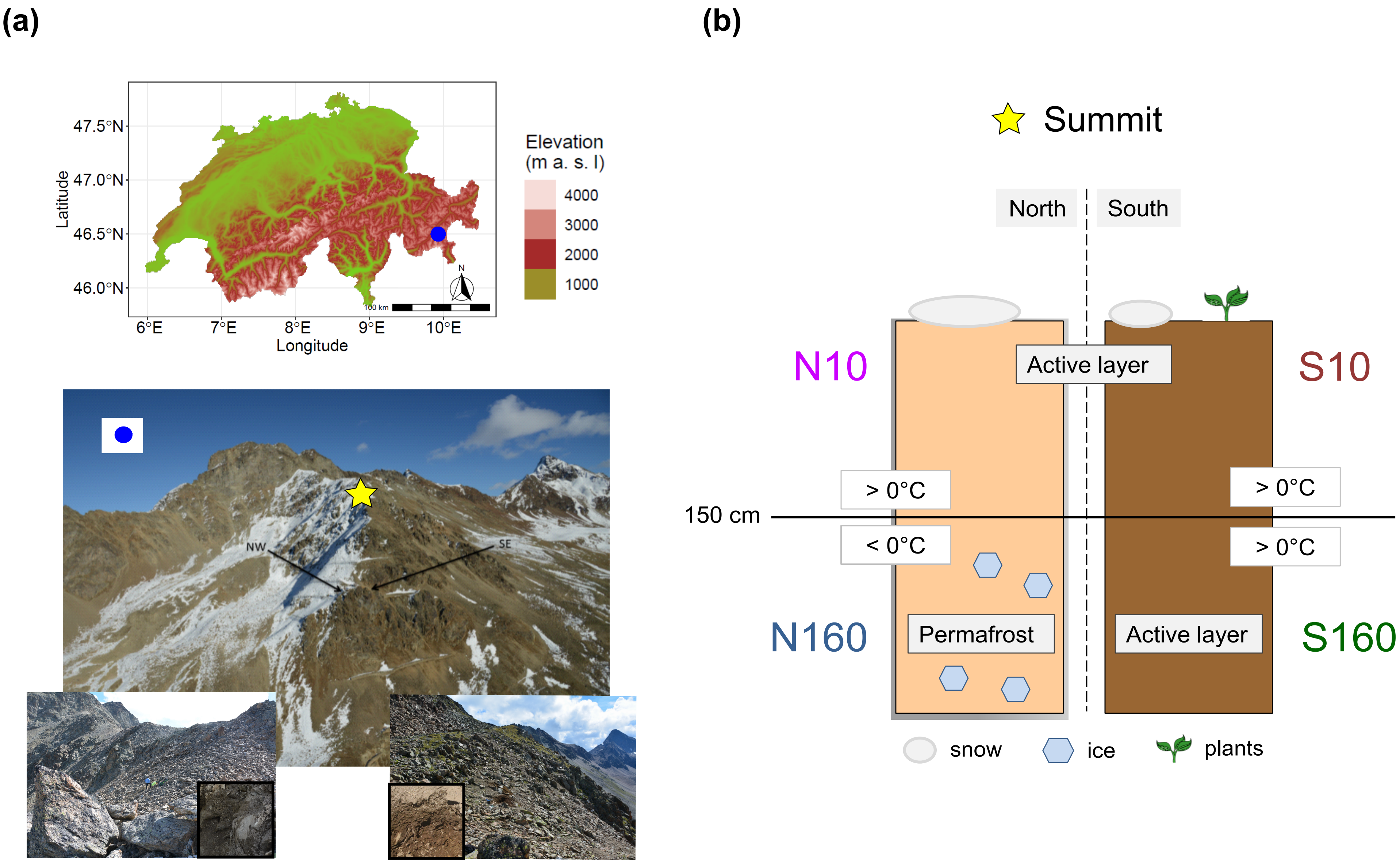
(a)
(b)
47.5°N
47.0°N
46.5°N
46.0°N
Latitude
6°E
7°E
8°E
9°E
10°E
Longitude
Elevation
(m a. s. l)
4000
3000
2000
1000
N
100 km
NW
SE
Summit
North
South
N10
S10
Active layer
> 0°C
150 cm
< 0°C
> 0°C
> 0°C
N160
Permafrost
Active layer
S160
snow
ice
plants

Fig. S2

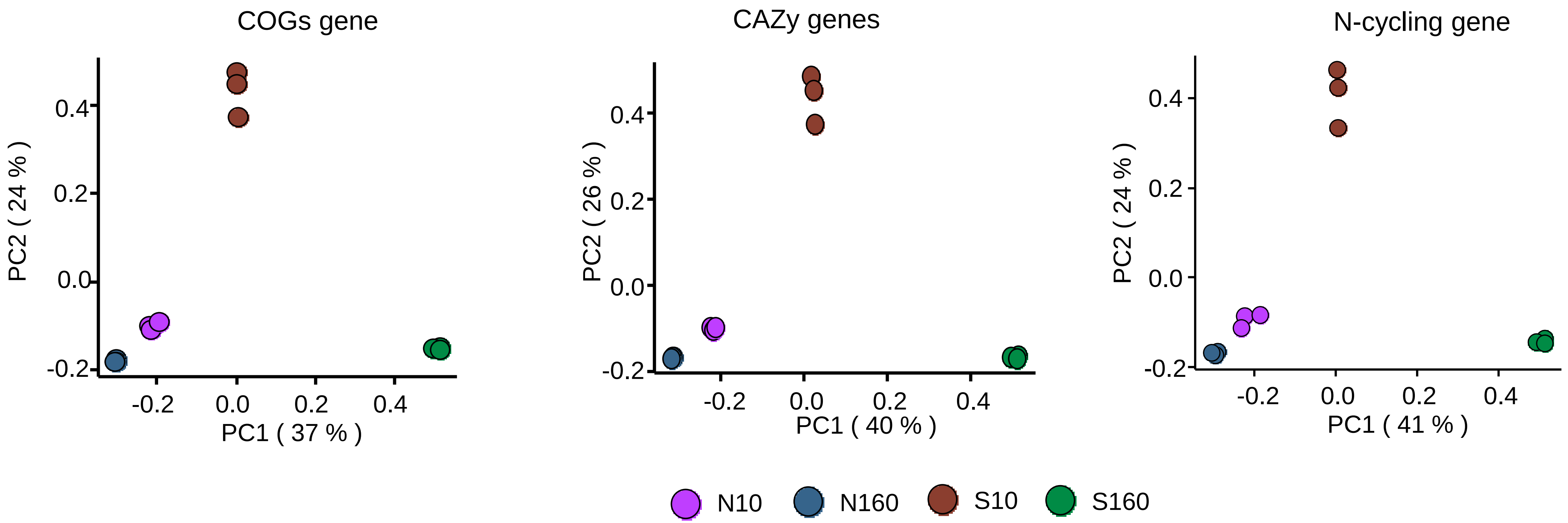

COGs gene
CAZy genes
N-cycling gene
PC2 (24 %)
PC2 (26 %)
PC2 (24 %)
0.4
0.2
0.0
-0.2
-0.2
0.0
0.2
0.4
PC1 (37 %)
PC1 (40 %)
PC1 (41 %)
N10
N160
S10
S160

Fig. S3

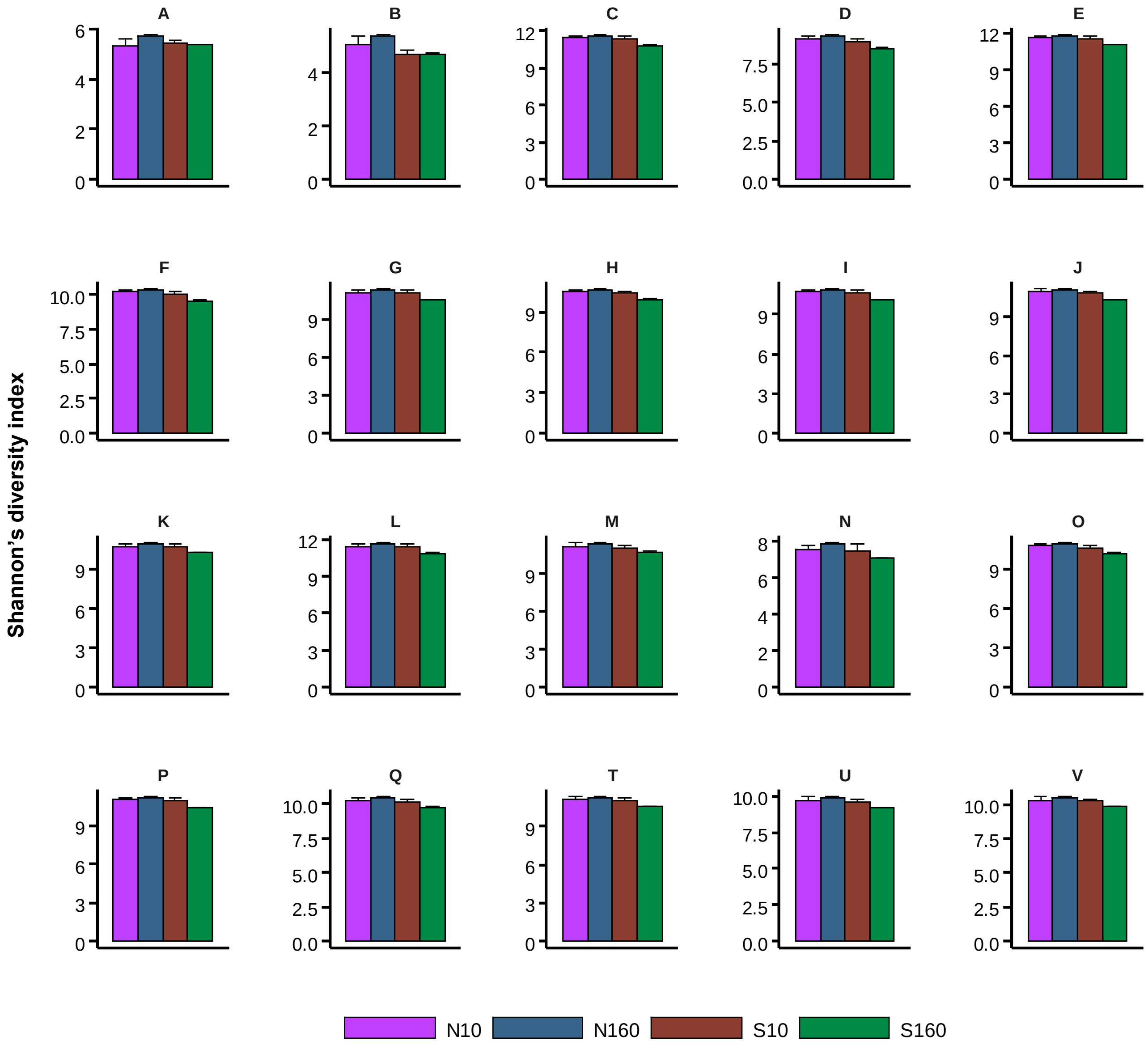

A RNA processing and modification
B Chromatin structure and dynamics
C Energy production and conversion
D Cell cycle control, cell division, chromosome partitioning
E Amino acid transport and metabolism
F Nucleotide transport and metabolism
G Carbohydrate transport and metabolism
H Coenzyme transport and metabolism
I Lipid transport and metabolism
J Translation, ribosomal structure and biogenesis
K Transcription
L Replication, recombination and repair
M Cell wall/membrane/envelope biogenesis
N Cell Motility
O Posttranslational modification, protein turnover, chaperones
P Inorganic ion transport and metabolism
Q Secondary metabolites biosynthesis, transport and catabolism
T Signal transduction mechanisms
U Intracellular trafficking, secretion, and vesicular transport
V Defense mechanisms

Fig. S4

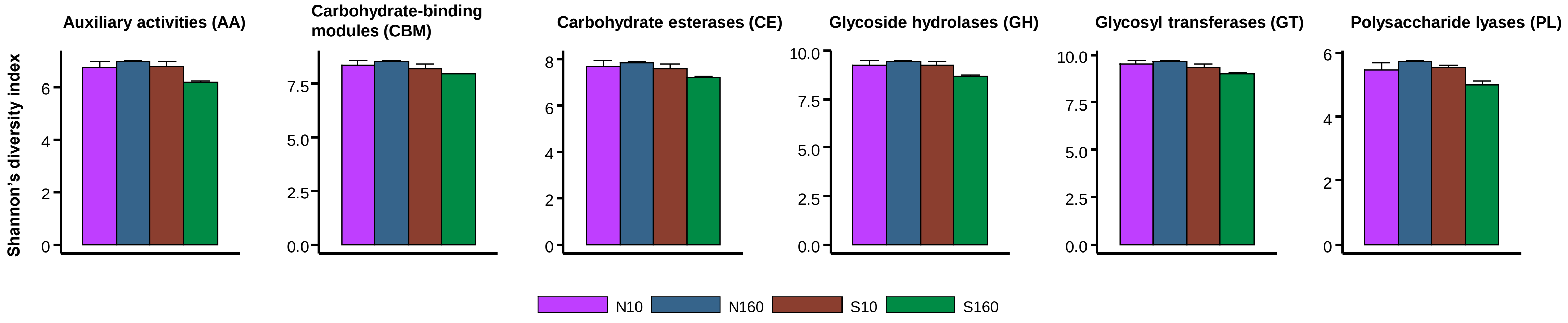

Auxiliary activities (AA)
Carbohydrate-binding modules (CBM)
Carbohydrate esterases (CE)
Glycoside hydrolases (GH)
Glycosyl transferases (GT)
Polysaccharide lyases (PL)
Shannon's diversity index
0
2
4
6
0.0
2.5
5.0
7.5
8
10.0
N10
N160
S10
S160

Fig. S5

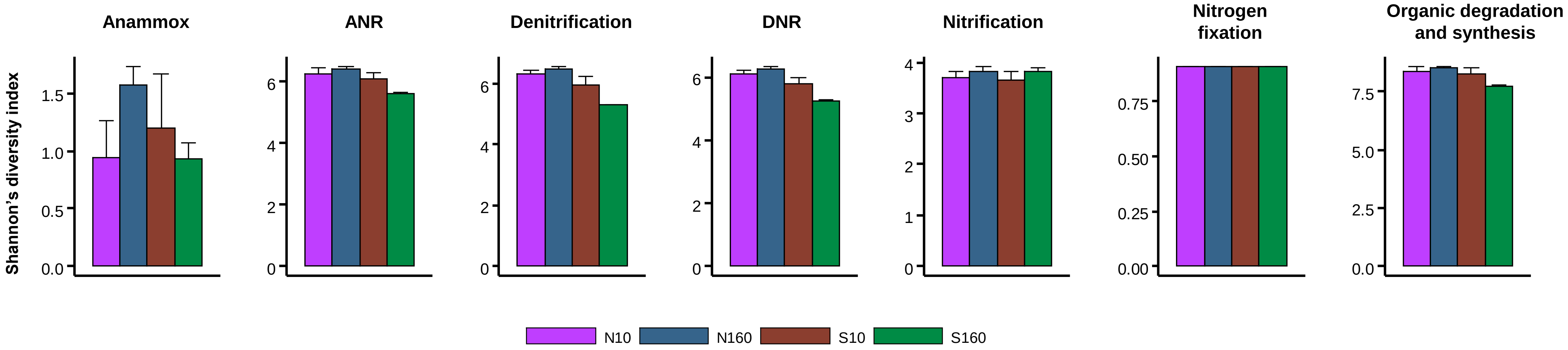

Anammox
ANR
Denitrification
DNR
Nitrification
Nitrogen fixation
Organic degradation and synthesis
Shannon's diversity index
0.0
0.5
1.0
1.5
0
2
4
6
0
1
2
3
4
0.00
0.25
0.50
0.75
0.0
2.5
5.0
7.5
N10
N160
S10
S160

Fig. S6

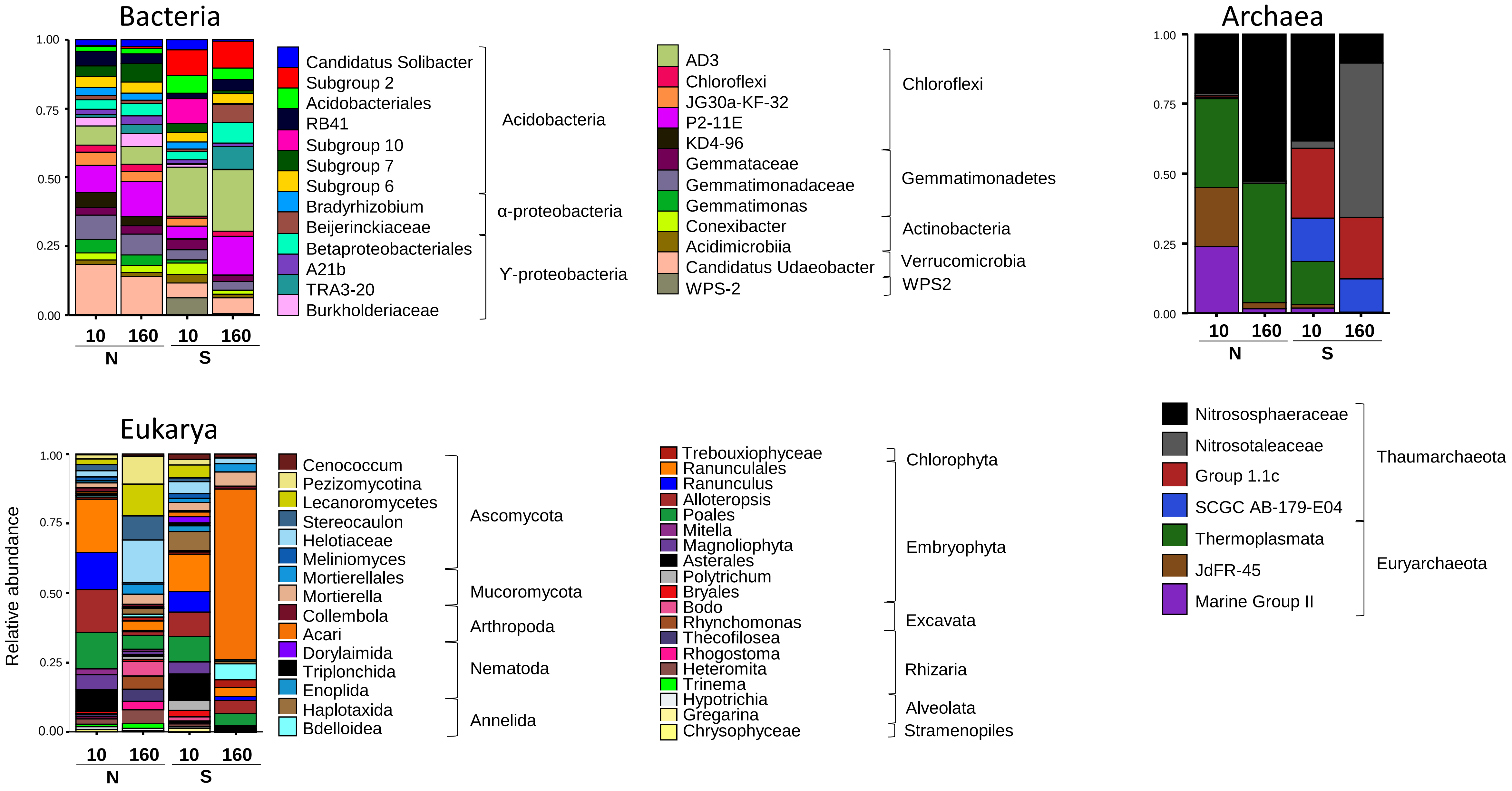

Bacteria
1.00
0.75
0.50
0.25
0.00
10
160
10
160
N
S
Candidatus Solibacter
Subgroup 2
Acidobacteriales
RB41
Subgroup 10
Subgroup 7
Subgroup 6
Bradyrhizobium
Beijerinckiaceae
Betaproteobacteriales
A21b
TRA3-20
Burkholderiaceae
Acidobacteria
α-proteobacteria
Ɣ-proteobacteria
AD3
Chloroflexi
JG30a-KF-32
P2-11E
KD4-96
Gemmataceae
Gemmatimonadaceae
Gemmatimonas
Conexibacter
Acidimicrobiia
Candidatus Udaeobacter
WPS-2
Chloroflexi
Gemmatimonadetes
Actinobacteria
Verrucomicrobia
WPS2
Archaea
1.00
0.75
0.50
0.25
0.00
10
160
10
160
N
S
Nitrososphaeraceae
Nitrosotaleaceae
Group 1.1c
SCGC AB-179-E04
Thermoplasmata
JdFR-45
Marine Group II
Thaumarchaeota
Euryarchaeota
Eukarya
Relative abundance
1.00
0.75
0.50
0.25
0.00
10
160
10
160
N
S
Cenococcum
Pezizomycotina
Lecanoromycetes
Stereocaulon
Helotiaceae
Meliniomyces
Mortierellales
Mortierella
Collembola
Acari
Dorylaimida
Triplonchida
Enoplida
Haplotaxida
Bdelloidea
Ascomycota
Mucoromycota
Arthropoda
Nematoda
Annelida
Trebouxiophyceae
Ranunculales
Ranunculus
Alloteropsis
Poales
Mitella
Magnoliophyta
Asterales
Polytrichum
Bryales
Bodo
Rhynchomonas
Thecofilosea
Rhogostoma
Heteromita
Trinema
Hypotrichia
Gregarina
Chrysophyceae
Chlorophyta
Embryophyta
Excavata
Rhizaria
Alveolata
Stramenopiles

Supplementary tables (together with its corresponding legend) are provided as single **Sheets** in an unique **xlsx** file.
